# Measuring hygiene competence: the picture-based situational judgement test HygiKo

**DOI:** 10.1186/s12909-021-02829-y

**Published:** 2021-07-30

**Authors:** Susanne Katharina Heininger, Maria Baumgartner, Fabian Zehner, Rainer Burgkart, Nina Söllner, Pascal O. Berberat, Martin Gartmeier

**Affiliations:** 1 Klinikum rechts der Isar, TUM Medical Education Center, Fakultät für Medizin, TU München, Ismaninger Straße 22, D-81675 München, Germany; 2 DIPF | Leibniz-Institut für Bildungsforschung und Bildungsinformation, Frankfurt, Germany; 3 Klinik und Poliklinik für Orthopädie und Sportorthopädie, Fakultät für Medizin, Klinikum rechts der Isar, TU München, München, Germany

**Keywords:** Situational judgement test, Hygiene, Competence, Assessment, Item-response theory

## Abstract

**Background:**

With the onset of the COVID-19 pandemic at the beginning of 2020, the crucial role of hygiene in healthcare settings has once again become very clear. For diagnostic and for didactic purposes, standardized and reliable tests suitable to assess the competencies involved in “working hygienically” are required. However, existing tests usually use self-report questionnaires, which are suboptimal for this purpose. In the present study, we introduce the newly developed, competence-oriented *HygiKo* test instrument focusing health-care professionals’ hygiene competence and report empirical evidence regarding its psychometric properties.

**Methods:**

HygiKo is a Situational Judgement Test (SJT) to assess hygiene competence. The HygiKo-test consists of twenty pictures (items), each item presents *only one unambiguous* hygiene lapse. For each item, test respondents are asked (1) whether they recognize a problem in the picture with respect to hygiene guidelines and, (2) if yes, to describe the problem in a short verbal response. Our sample comprised *n* = 149 health care professionals (79.1 % female; age: *M* = 26.7 years, *SD* = 7.3 years) working as clinicians or nurses. The written responses were rated by two independent raters with high agreement (α > 0.80), indicating high reliability of the measurement. We used Item Response Theory (IRT) for further data analysis.

**Results:**

We report IRT analyses that show that the HygiKo-test is suitable to assess hygiene competence and that it allows to distinguish between persons demonstrating different levels of ability for seventeen of the twenty items), especially for the range of low to medium person abilities. Hence, the HygiKo-SJT is suitable to get a reliable and competence-oriented measure for hygiene-competence.

**Conclusions:**

In its present form, the HygiKo-test can be used to assess the hygiene competence of medical students, medical doctors, nurses and trainee nurses in cross-sectional measurements. In order to broaden the difficulty spectrum of the current test, additional test items with higher difficulty should be developed. The Situational Judgement Test designed to assess hygiene competence can be helpful in *testing* and *teaching* the ability of working hygienically. Further research for validity is needed.

## Background

Nosocomial infections are a serious challenge in modern patient care [1] and have recently been subject of intense research [2]. In order to prevent hospital-acquired infections (HAI) and improve patient and staff safety, hygiene is essential. In pandemics like COVID-19 which started in the year 2019, the prevention of infections through consequent implementation of hygiene measures is crucial. Some existing intervention formats are designed to increase awareness and improve attitudes towards hygiene in daily clinical work [3–5]. While there are numerous single studies (e.g. [6], [7, 8]) and a number of meta-analyses [9, 10] on the issue of hygiene, these mostly focus on interventions for improving *hand* hygiene. A massive international campaign for the prevention of infection, the “My Five Moments of Hand Hygiene”-program [11], also puts the emphasis on hand hygiene. We argue, however, that the focus on hand hygiene is too narrow because health care staff draw upon a differentiated repertoire of cognitive resources in order to adhere to hygiene standards in daily work. This argument is supported by the *WHO Guidelines on Core Components of Infection Prevention and Control (IPC) Programmes* [12]. These guidelines, based on the *WHO Core Components for Infection Prevention and Control Report*, were extended in 2016, highlighting additional areas for preventing infections in health care facilities worldwide, especially in acute health care facilities: personnel and facility resources, including workload, staffing, materials and equipment (guideline recommendations numbers seven and eight). This points out the need to develop a more differentiated concept of hygiene competence – which also requires more sophisticated measurement strategies.

In a recent article, Gartmeier et al. [13] proposed a multidimensional model of *hygiene competence* consisting of three dimensions, *knowledge*, *skills* and *attitudes*. They argue that *knowledge* about why hygiene is important and how it can be maintained in specific clinical situations is necessary, but not sufficient. Moreover, health care staff need specific *skills* in order to apply their knowledge in hygienic patient care. Finally, specific *attitudes* are required, e.g. maintaining hygienic working procedures despite high time pressures and understaffing. What this model proposes (especially compared to more general models such as the “My Five Moments of Hand Hygiene” [11]) is that in order to uphold hygiene across a multitude of particular health care tasks (e.g., providing artificial respiration or replacing a patient’s bladder catheter), specific procedures are performed which require synergy between all three dimensions of hygiene [13] described above.

Based on this more comprehensive understanding of hygiene as a professional competence, we argue that psychometrically promising instruments should be designed to assess this competence. For this purpose, the use of Item Response Theory enables us to develop innovative assessment instruments with all capabilities modern test theory has to offer; for example, more sophisticated ability estimation and sample-independent item calibration, more flexible test designs such as adaptive testing and many others. The approach to measurement should take account of the fact that health care staff manage to synthesize different resources – knowledge, skills and attitudes – in order to maintain hygiene in their daily work practice. We propose that a *Situational Judgement Test* (SJT) requiring test respondents to identify the presence or absence of hygiene problems in realistic images of clinical situations is a promising approach in this respect. We elaborate this conjecture in what follows.

### Assessment of hygiene competence

Currently, researchers primarily use self-report questionnaires to assess hygiene-related constructs with regard to attitudes and practices (see e.g. [6] [14]). Evidence shows, however, that such instruments are suboptimal in measuring competencies [15], partly because health care staff systematically overestimate the extent of hygiene-related behaviors they perform in clinical practice [16]. More objective measures should therefore be developed to assess hygiene competence. In order to measure competencies, direct observations of clinical practice as well as realistic simulations of such practice are optimal, but are resource-intensive and cannot be easily standardized [17, 18]. With regard to hand hygiene, different observational tools are available [19–21], but no current instrument adopts the more comprehensive measurement approach suggested by the above mentioned model of hygiene competence [13]. Drawing upon this model, we propose that a SJT is a promising, time- and cost-efficient method of assessment. SJTs require respondents to make knowledge-based judgements of scenarios displayed as short texts, pictures or videos [22] [23]. Properly designed SJTs yield reliable measures, they are good predictors of job performance and are well accepted by test respondents [23]. Moreover, SJTs allow assessments to be conducted in a standardized way – which is less of a drain on scarce resources and time [22, 23]. Such instruments are hence increasingly used in the medical context, for instance, in context of medical admission procedures [22–24]. Regarding the specifics of their design, current SJTs can „differ markedly from each other (in scenario content and response formats, for example)“. Patterson et al. (2016, [23]) advance that SJTs use dilemma-situations, but other tests have been published as SJTs which do not use dilemma-situations – e.g. the SJTs introduced by Kiessling et al. (2016) or by Pangallo et al. (2016).

In the Methods section below, we describe the design of our hygiene competence (HygiKo)-SJT which has been designed to measure the competence-dimensions *knowledge* and *practices/skills.* The HygiKo-SJT uses hypothetical scenarios respondents are likely to encounter in their clinical work (which is a typcial attribute of SJTs described by Patterson et al. (2016, [23], cf. p. 4). The HygiKo-test requires respondents to make knowledge-based judgements of still-images of common clinical work procedures and situations. Respondents have to apply their knowledge in order to judge whether the clinical practices shown in the images are carried out in compliance with hygiene standards. This assessment strategy relates to the second level in Miller’s widely used prismatic model of clinical competence [17], which represents “knows how” as the (cognitive) application of knowledge. It has been propsed that one characteristic of SJTs is that they measure non-academic attributes of healthcare professionals (e.g. empathy) [23]. We argue that the character of the HygiKo-SJT is in line with this description: What is often taught in primary medical education regarding the basics of hospital hygiene comes from the academic fields of immunology, infectiology or microbiology. However, for medical practitioners, the question is how they can put this knowledge into practice and perform their job in a way which does not endanger themselves or their patient because of deficits in hygiene. For this reason, we argue in favour of our SJT measuring a *non-academic attribute of healthcare*, just as Patterson et al. (2016, [23]) put it.

We will report a pilot study with an initial version of the HygiKo-SJT, focusing upon two research questions: (RQ1) Does the HygiKo-test comply with competence test quality requirements described in context of Item Response Theory (IRT)? (RQ2) Is it possible to cover a broad spectrum of item difficulty / person ability regarding hygiene competence by means of the HygiKo-SJT? Both research questions aim at checking psychometric requirements that are pivotal for a test’s quality. In particular, RQ2 focuses an important matter because the information yielded by psychometric tests is most informative (i.e., is affected least by error in ability estimation) in those regions of the ability continuum that the test’s item difficulties cover. That is, a test with low difficulty cannot measure differences between experts. Such a test might be well suited, e.g., for a population of novices starting their vocational education. With the HygiKo-SJT’s goal to be applicable to both, experienced practitioners as well as students or practitioners in training, it is desirable to sufficiently cover the entire ability continuum.

## Methods

### SJT design and measurement strategy

**Fig. 1:**
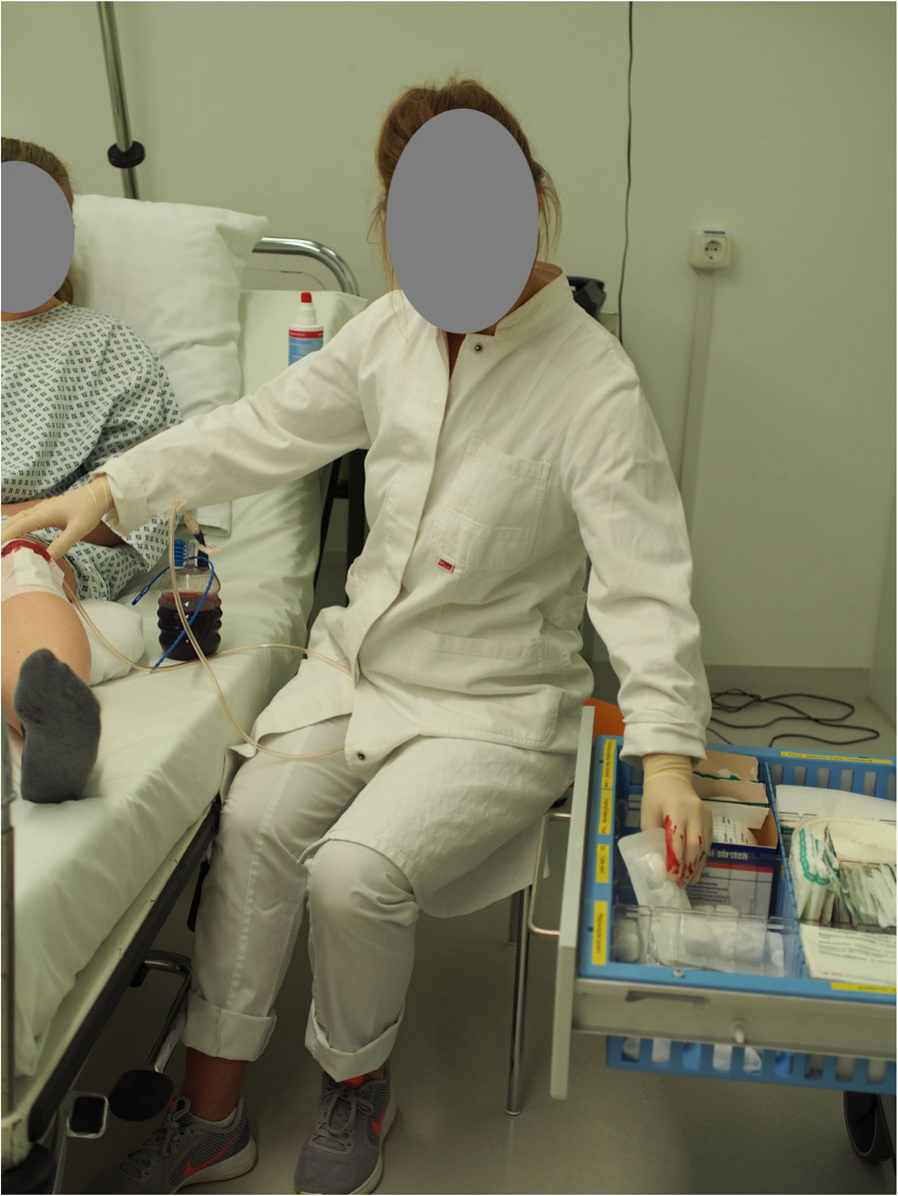
Sample item

The HygiKo-SJT is designed to assess hygiene competence and consists of twenty picture vignettes (see Fig. 1 for a sample item). Each vignette shows at least one health care provider (nurse [f/m] or clinician) and a patient (where appropriate) in a clinical situation in which hygiene is a relevant issue. The test items were constructed in the following way: Every item shows only one unambiguous hygiene-related fault (two items show no fault). The situations displayed (medical equipment and rooms) are authentic reconstructions of clinical situations. The respective photos were taken in different rooms (OR, patient room, treatment room, intensive care room) of the Medical Training Center (MTC) of the München rechts der Isar (MRI) university hospital. The situations shown in the test items represent clinical procedures performed by physicians and nurses (f/m; see Table 1). The reason for this interdisciplinary setup of HygiKo lies in the fact that hygiene competence often needs to be applied in collaborative settings. A thorough situation-specific understanding of hygiene in healthcare is therefore important regardless of healthcare profession. As described above, the test items refer to the hygiene model dimensions *knowledge* and *skills* [13]. The *attitudes*-dimension of the competence model could not directly be represented in the test. All persons depicted in the HygiKo-SJT items were non-professional actors from whom full consent was obtained. Materials, rooms, equipment and procedures were pictured as realistically as possible (e.g. picture vignettes showing a situation in OR were taken in a simulation OR with original materials). All situations displayed were conceptualized in close cooperation with and reviewed by nurses and medical doctors with extensive clinical experience to ensure a realistic portrayal of situations in the pictures.

### Sample item

The generated picture vignettes were compiled into a paper-pencil questionnaire. For each vignette, respondents were asked (1) to take down whether they recognize a problem in the picture regarding hygiene guidelines (dichotomous: yes/no) and (2), if yes, to describe the problem in a short verbal response. For each item, an ideal solution was developed based on scientific literature and national clinical hygiene guidelines. All picture vignettes and solutions were reviewed in cooperation with the group of local hygiene experts (who were advanced clinicians, nurses [f/m] and specialists from the local hygiene department). Only *completely correct* answers were credited if (1) the respondent recognized an incorrect (unhygienic) action in the picture *and* (2) *correctly* described the hygiene lapse (matching to the model solution). In contrast, respondents were not credited if they just recognized the incorrect (unhygienic) action, but could not point out what exactly was wrong (e.g. sterile gloves missing). In the present study, three raters (two pairs: raters A + B, raters B + C) evaluated all given answers and took a pass-fail-decision (dichotomous) based on the model solution. Krippendorff’s alpha [25] was calculated to measure the inter-rater reliability of the rating procedure.

**Table 1:** Content of the HygiKo-SJT test items

Item	Content / Issue	Hygiene problem
1	Incorrect wearing of surgical face mask	Yes
2	Incorrect hand posture during pre-surgery dressing	Yes
3	Nurse incorrectly standing next to a sterile table	Yes
4	Wearing of personal items (wristwatch) at bedside	Yes
5	Personal sluice (incorrect changing of personal to work clothing)	Yes
6	Non-sterile suctioning of mucus	Yes
7	Putting blood sampling set down on patient’s bedside	Yes
8	Personal hygiene – nurse’s hair touching patients bed	Yes
9	Sterile hand gloves being transported in nurse’s dress pocket	Yes
10	Personal hygiene – tie touching patient’s bed blanket	Yes
11	Personal hygiene (artificial fingernails) in clinical practice	Yes
12	Reaching into care trolley drawer with contaminated hand gloves	Yes
13	Using care trolley in isolated patient room	Yes
14	Wearing medical work clothes in public areas	Yes
15	No-touch-technique	No
16	Hygienically transporting medical file on patient bed	No
17	Typing on computer keyboard wearing sterile hand gloves	Yes
18	Carrying infusion bottle in nurse’s dress pocket	Yes
19	Opening sterile syringe packaging	Yes
20	Wearing personal items (jewelry) in clinical practice	Yes

### Sample

The sample was recruited from various compulsory seminars of the medical studies, from an introductory event for the practical year after graduation, from experienced physicians as well as from volunteer nurses and nursing students. The sample comprised *n* = 149 health care professionals (79.1 % female; age: *M* = 26.7 years, *SD* = 7.3 years) (f/m; see Table 2 for overview).

**Table 2:** Professions and degrees of professional experience in the study sample

Profession	Frequency *in %*	Professional Experience in Years	
		*M* (*SD*)	Freq. *in %*
Medical Student	40.8	5.6 (0.85)	
Medical Doctor	4.8	7.0 (8.49)	
Trainee Nurse^a^ (f/m)	34.7	2.2 (1.00)	First year: 41.7
			Third year: 58.3
Nurse (f/m)	19.0	14.3 (13.81)	
Trainee operating room technician (ORT)^b^	0.7	1.0 (0)	First year: 100

^a^ In Germany, nursing education is a vocational college training, which is not rooted at a university. Trainee nurses spent three years at a vocational school for theoretical and practical education (2,100 h). Additionally, trainee nurses serve 2,500 h at a teaching hospital for practical training

^b^ In Germany, ORT education is based at a vocational college as well. The theoretical education covers 1,600 h, the practical training includes 3,000 h at a teaching hospital

### Analysis

We used Item Response Theory (IRT) for data analysis. The IRT framework provides a family of probabilistic models, which allow for simultaneous estimation of respondent ability and item difficulty [26]. This way, we could model “the interaction between an individual item and an individual examinee” [27] by using the Rasch Model, the most restrictive IRT model with the desirable measurement properties. Only if a test has been shown to produce data conforming to the Rasch Model is it fair to use the sum score. This model can be used to estimate unidimensional latent abilities [26]. There is a discussion about the use of a Rasch Model in SJTs (please see Tiffin et al. 2019: “The cross-cutting edge: situational judgement tests for selection: traditional versus construct-driven approaches”). Still, we decided to use this combination on basis of the reasons mentioned above. The Rasch Model assumes that the probability *P*(X_*v,i*_) of a person *ν* giving a certain answer *X* for an item *i* depends on two factors: (1) the item difficulty, σ_*i*_, and (2) the person’s ability θ_*ν*_ [28], which are estimated simultaneously on the same scale. This means, the numbers for person abilities and item difficulties can be directly compared. If a person with ability θ_v_ = 1.1 responds to an item with difficulty σ_*i*_ = 1.1, the probability of a correct response is 50 %. But if the difference between ability and difficulty is largely positive or negative, the probability of a correct response gets close to 100 or 0 %, respectively. Negative parameter values indicate simple items or low ability; positive values refer to difficult items or high ability [29]. Mathematically, these relationships are modelled by the following equation:

$$P\left({X}_{i,v}=1 | {{{\upsigma }}_{i},{\uptheta }}_{v}\right)= \frac{\text{e}\text{x}\text{p}({{\uptheta }}_{v}-{{\upsigma }}_{i})}{1+\text{e}\text{x}\text{p}({{\uptheta }}_{v}-{{\upsigma }}_{i})}$$

In order to test whether collected data are compliant with the Rasch Model, it is crucial to confirm the model’s assumption of *specific objectivity* [26]. This means that different subpopulations in a sample should not differ significantly regarding item difficulties. To measure this, it is common to split a sample into subsamples and compare the resulting item difficulties using the *Andersen Likelihood Ratio Test* [30]. Since the null hypothesis assumes no differences, it is common to use three split criteria, and to apply a Type-I-risk of α = 0.01, so this risk inflates to no more than α = 0.05 across all comparisons [26]. In this study, we used the split criteria *raw score median* (comparing two achievement groups), *gender* and *age median*. IRT analyses were carried out using the R packages *eRm* and *PP* [31–33].

## Results

### Inter-rater-reliability

We calculated Krippendorff’s alpha for inter-rater reliablity and found strong agreement (α ≥ 0.80; see Table 3).

**Table 3:** Krippendorff’s alpha for rater sets

Rater Group	Krippendorff’s alpha
Raters A + B	0.81
Raters B + C	0.86

*Note*. Rater A: student rater; rater B/C: research associates

### IRT Scaling (research questions one and two)

**Fig. 2:**
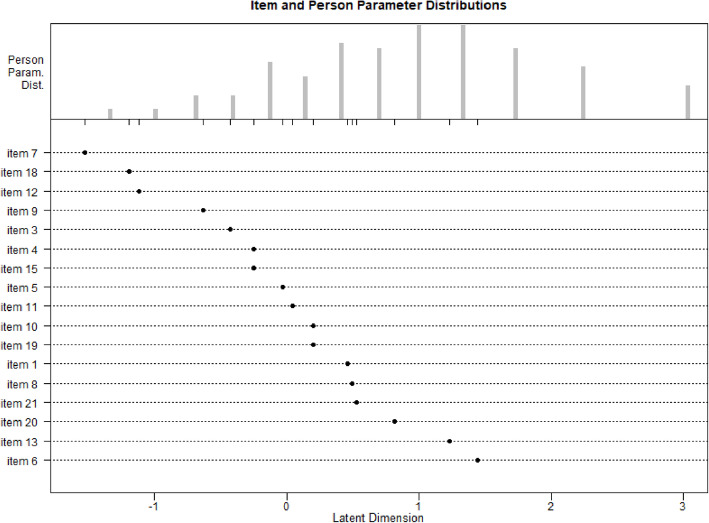
Person ability (top) and item parameter (bottom) distribution for the HygiKo-SJT

Based on Andersen’s Likelihood Ratio Test, three of the twenty items of the HygiKo-SJT had to be excluded in order to reach sufficient model fit. Using the remaining seventeen items, Rasch Model conformity could be assumed for the instrument – see test statistics displayed in Table 4. The Wald test indicated which item had to be excluded. Figure 2 shows the resulting item difficulty parameters and person parameter distributions. An item’s measurement is most informative if its difficulty is about equal to a person’s ability.

**Table 4:** Rasch Model Conformity Achieved for the Final Scale with 17 Items

Split Criterion	LRT χ²	df	χ²_df,α=0.01_	*p*
High / Low Achievement	28.48	16	32.00	0.028
Female / Male	31.81	16	32.00	0.011
Younger / Older	31.87	16	32.00	0.010

As is apparent, the HygiKo test items cover a difficulty spectrum from low to medium; no item showed a high degree of difficulty. Some test respondents, however, showed higher ability, meaning they were seemingly able to solve all test items (cf. upper section of cf. Fig. 2).

## Discussion

In this paper, we have described the HygiKo-SJT, a novel, time and cost-efficient, yet psychometrically promising method of assessing hygiene competence based upon a theoretical model [13]. The HygiKo-SJT, originally consisting of twenty picture items, offers a feasible way of assessing hygiene competence. The responses given by the study participants were rated by two independent raters with high agreement (α > 0.80), indicating the high reliability of the measurement. Further, the reported IRT analyses show that the HygiKo-SJT is suitable to assess hygiene competence and that it is possible to distinguish between persons with different levels of ability, independent of the respective profession. After exclusion of three items, the remaining seventeen items showed good measurement quality (RQ1). Further, the results showed that our test items cover the range of low to medium person ability regarding hygiene competence. The current results show that the initial test version does not contain items with sufficient difficulty to discriminate for very high abilities (i.e., highly able test respondents would answer all items correctly, RQ2). Nevertheless, because we did not observe a strong ceiling effect, the test in its present form can be used to assess hygiene competence of medical students, medical doctors, nurses and trainee nurses in cross-sectional measurements.

Besides the SJT introduced here, other instruments exist which focus hygiene-related attitudes or knowledge of employees in health care settings (e.g. [6, 14]). We argue that our instrument amends the current state of research in this context for three reasons:

First, our measurement approach is less prone to known biases of self-report instruments, like social desirability. This is because to make correct judgements in our SJT, one needs to have knowledge about hygiene-related practices (e.g. [6]). This not the case with existing instruments focusing attitudes regarding hygiene. Second, existing instruments focus health care workers knowledge about hospital hygiene (e.g. World Health Organization (WHO) Hand Hygiene Knowledge Questionnaire (revised 2009). A disadvantage of such instruments is that in clinical practice, having detailed knowledge is not sufficient in order to work hygienically, the knowledge needs to be applied correctly across a multitude of situations. Our SJT requires professionals to apply their hygiene-knowledge to concrete situations visualized as pictures in order to judge these situations. Of course, this is not the same as doing clinical work in accordance with hygiene standards. However, we argue that the test approach of the HygiKo-SJT provides a relevant approximation of this competence and hence valuably amends existing empirical approaches in this respect. However, further research is surely necessary in order to investigate this conjecture. Third, we argue that our study adds to the existing literature because it amends our understanding of what constructs SJTs are suitable to measure. Patterson et al. (2016, [23] cf. p. 3) advance that SJT are useful to measure “prosocial Implicit Trait Policies (ITPs)”. Hygiene competence, which is the focus of our SJT, surely falls outside the area of such policies.

A general conclusion to be drawn from the present results is that the described measurement strategy offers a promising means of estimating professionals’ hygiene competence. Nevertheless, a critical remark has to be made regarding the HygiKo-SJT in its current version: The HygiKo-SJT covers a broad range of clinical situations, some of which may be regarded as special cases (e.g. items 1, 2, 3 and 5 showing situations in the OR). For this reason, it could be argued that these items are very difficult to answer for respondents who have no or only very limited experience regarding this very specific area of health care. However, as is apparent from Table 1, the operating-room items 1, 3 and 5 all had medium difficulty levels. Thus, these outcomes *do not suggest* that respondents had substantial difficulties in answering these items because they were not familiar with these situations.

In order to further develop the HygiKo-SJT, several perspectives are recommended: First, we suggest a data-based strategy to determine what is measured by the SJT. To achieve this, correlations with data collected with existing hygiene-related measures (e.g. [6] [14]) should be examined. In addition, investigating which correlations can be detected between the measure of hygiene competence obtained by the HygiKo-SJT and performance in simulated situations or even in clinical practice would be useful. In order to broaden the difficulty spectrum of the HygiKo-SJT, additional test items with higher difficulty could be developed. This can be achieved by using more specific situations as basis of test-items, e.g. in the OR, with special treatment situations or involving multiple professional actors. An alternative way would be to use more complex item formats, such as short videos, instead of picture vignettes. In videos, hygiene lapses could be shown only for a short time interval, making the measurement even more authentic by mirroring the dynamic nature of clinical situations as they occur in real-world. However, the additional use of video items would maybe also require investigating the attention processes of the test respondents. As video items would allow complex reality to be presented with the options of stopping or slowing the playback, such items could also be particularly useful for *teaching* hygiene competence. In an extended version in the future of the hygiene test, is it conceivable that a part for survey the attitudes next to knowledge and skills as the three parts of the hygiene competence model according to Gartmeier et al. [13] could also be added. For the first version, we wanted to focus on measuring the competence not possibly confounded by personality traits.

## Conclusions

Since the onset of the COVID-19 pandemic at the beginning of 2019, the high importance of hygiene has become clear to everyone in the health care sector and beyond. Due to this momentum, teaching hygiene competence to medical students may receive even greater attention than before. In order to determine whether respective courses contribute to competence development, suitable test formats are required which allow for testing newly acquired hygiene-related competencies. The HygiKo-SJT is designed to assess hygiene competence and may thus be helpful to address the urgent need to teach and test the ability to work hygienically in the medical context.

## Data Availability

All data and materials described here are available from the corresponding author upon request.
