# Correction to: Measuring hygiene competence: the picture-based situational judgement test HygiKo

**DOI:** 10.1186/s12909-021-02877-4

**Published:** 2021-08-19

**Authors:** Susanne Katharina Heininger, Maria Baumgartner, Fabian Zehner, Rainer Burgkart, Nina Söllner, Pascal O. Berberat, Martin Gartmeier

**Affiliations:** 1 Klinikum rechts der Isar, TUM Medical Education Center, Fakultät für Medizin, TU München, Ismaninger Straße 22, D-81675 Munich, Germany; 2 DIPF | Leibniz-Institut für Bildungsforschung und Bildungsinformation, Frankfurt, Germany; 3 Klinik und Poliklinik für Orthopädie und Sportorthopädie, Fakultät für Medizin, Klinikum rechts der Isar, TU München, Munich, Germany

**Correction to: BMC Medical Education 21, 410 (2021)**

**https://doi.org/10.1186/s12909-021-02829-y**

Following publication of the original article [1], the authors identified and would like to correct:

The explaining text below the sample vignette incorrectly reads *„Fig. 2 Person ability (top) and item parameter (bottom) distribution for the HygiKo-SJT* “instead of *„Fig. 1 Sample item “and* vice versa*.*

The correct descriptions to Figs. 1 and 2 are:

- *„Figure 1 Sample item “(page 4 of 8)*
- *“Figure 2 Person ability (top) and item parameter (bottom) distribution for the HygiKo-SJT “(page 6 of 8)*

The original article has been corrected.
